# Association of Urinary Pentosidine Levels With the Risk of Fractures in Patients With Severe Osteoporosis: The Japanese Osteoporosis Intervention Trial‐05 (JOINT‐05)

**DOI:** 10.1002/jbm4.10673

**Published:** 2022-08-31

**Authors:** Shiro Tanaka, Mitsuru Saito, Hiroshi Hagino, Satoshi Mori, Toshitaka Nakamura, Hiroaki Ohta, Teruki Sone, Kaito Takahashi, Yuji Mitomo, Toshitsugu Sugimoto, Satoshi Soen, Adequate Treatment of Osteoporosis (A‐TOP) Research Group

**Affiliations:** ^1^ Department of Clinical Biostatistics Graduate School of Medicine Kyoto University Kyoto Japan; ^2^ Department of Orthopedic Surgery Jikei University School of Medicine Tokyo Japan; ^3^ School of Health Science Tottori University Faculty of Medicine Yonago Japan; ^4^ Seirei Hamamatsu General Hospital Hamamatsu Japan; ^5^ Touto Sangenjaya Rehabilitation Hospital Tokyo Japan; ^6^ Department of Obstetrics and Gynecology 2 Kawasaki Medical School Okayama Japan; ^7^ Department of Nuclear Medicine Kawasaki Medical School Kurashiki Japan; ^8^ Eikokai Ono Hospital Ono Japan; ^9^ Soen Orthopaedics Osteoporosis and Rheumatology Clinic Kobe‐shi Japan

**Keywords:** AGING, BIOCHEMICAL MARKERS OF BONE TURNOVER, BONE MATRIX, CLINICAL TRIALS, EPIDEMIOLOGY

## Abstract

Associations between urinary pentosidine, one of the advanced glycation end products in collagen, and the risk of fracture in patients with severe osteoporosis are unknown. In this study, we investigated whether the urinary pentosidine level is associated with the incidence of morphometric vertebral fracture and nonvertebral fracture using data of a randomized, controlled trial, JOINT‐05. JOINT‐05 enrolled Japanese women aged 75 years or older with primary osteoporosis. Patients were randomly assigned (1:1) to receive sequential therapy (teriparatide followed by alendronate) or monotherapy with alendronate for 120 weeks. Incidences of vertebral and nonvertebral fractures were assessed morphologically. During treatment, urinary levels of pentosidine and serum levels of bone turnover markers (osteocalcin, procollagen type I amino‐terminal propeptide, and tartrate‐resistant acid phosphatase 5b) were measured. A total of 967 patients with baseline pentosidine levels were included in the study. Of these, 137 had vertebral fractures, and 42 had nonvertebral fractures. The rate ratios for vertebral fracture for the second (30–39 pmol/mL), third (40–49 pmol/mL), and fourth quartile (≥50 pmol/mL) groups divided by pentosidine level compared with the first quartile (<30 pmol/mL) group were 1.65 (95% confidence interval [CI] 0.99–2.75, *p* = 0.06), 1.51 (95% CI 0.87–2.61, *p* = 0.14), and 1.69 (95% CI 1.01–2.83, *p* = 0.05), respectively. The corresponding rate ratios for nonvertebral fracture were 3.07 (95% CI 0.88–10.70, *p* = 0.08), 2.34 (95% CI 0.61–8.95, *p* = 0.22), and 3.95 (95% CI 1.14–13.67, *p* = 0.03), respectively. The association of the urinary pentosidine level with the incidence of nonvertebral fracture was the strongest among the biomarkers assessed in the study. In conclusion, the urinary pentosidine level was associated with the risk of fracture in patients with severe osteoporosis receiving teriparatide or alendronate. © 2022 The Authors. *JBMR Plus* published by Wiley Periodicals LLC on behalf of American Society for Bone and Mineral Research.

## 1 Introduction

With the development of new therapeutic agents for osteoporosis, such as teriparatide, romosozumab, and denosumab, assessment of fracture risk during treatment is becoming increasingly important. The Endocrine Society guideline has adopted an algorithm for treatment selection on the basis of the 10‐year fracture probability.^(^ ^1^ ^)^ The National Institute for Health and Care Excellence of the United Kingdom (UK) also issued a guideline^(^ ^2^ ^)^ stating that reassessment of fracture risk using FRAX^(^ ^3^ ^)^ or QFracture^(^ ^4^ ^)^ is important to decide whether a “drug holiday” is needed for adults receiving osteoporosis medication for a long time. However, these fracture risk assessment tools were developed based on the results of population‐based cohort studies in treatment‐naïve subjects, and their accuracy for prediction in patients on pharmacological treatment is unclear. Thus, the UK guideline points out that prospective studies investigating the predictive power of these tools to assess fracture risk in treated patients is necessary.^(^ ^2^ ^)^ In recent years, several biomarkers of bone turnover^(^ ^5^ ^)^ or bone quality^(^ ^6^ ^)^ have been developed, and new clinical risk factors including comorbidities,^(^ ^4^ ^)^ genes,^(^ ^7^ ^)^ and nutrition^(^ ^8^ ^)^ have been reported, but these biomarkers or clinical factors are not considered in the existing fracture risk assessment tools.^(^ ^3^, ^4^, ^9^ ^)^ Because fracture risk is conceptually determined by bone quantity and quality, bone quality markers may contribute to risk assessment during the treatment of osteoporosis.

Pentosidine, one of the advanced glycation end products (AGEs) in collagen, is a senescent nonenzymatic cross‐link resulting from glycation and oxidation.^(^ ^10^ ^)^ Accumulation of pentosidine impairs the mechanical properties of bone and is associated with brittleness of collagen fibers.^(^ ^11^, ^12^, ^13^, ^14^ ^)^ In previous cohort studies, urinary pentosidine levels were associated with the incidence of fracture in postmenopausal women^(^ ^15^, ^16^ ^)^ or patients with type 2 diabetes mellitus.^(^ ^16^ ^)^ The univariate analysis of the OFELY Study also showed similar associations, although the results were not statistically significant after adjustment for age, bone mineral density (BMD), and prevalent fracture.^(^ ^17^ ^)^ In the Nagano Cohort Study, urinary pentosidine levels were quantified by a conventional method and a new method, namely, the high‐performance liquid chromatography (HPLC) method,^(^ ^15^, ^18^, ^19^ ^)^ and the enzyme‐linked immunosorbent assay (ELISA) method, respectively.^(^ ^20^ ^)^ The ELISA method is more convenient than the HPLC method because it does not require sample pretreatment with heat hydrolysis, and a recent study reported a cross‐sectional association between urinary pentosidine levels measured by the ELISA method and prevalent fracture.^(^ ^20^ ^)^ This accumulated evidence suggests the potential of urinary pentosidine to be a risk factor for fracture, but data on treated patients are sparse.

Therefore, a retrospective, cohort study was performed to investigate whether the urinary pentosidine level is associated with the incidence of morphometric vertebral fracture and nonvertebral fracture. The associations between fracture incidence and the levels of the following bone turnover markers were also investigated: osteocalcin, procollagen type I amino‐terminal propeptide (P1NP), and tartrate‐resistant acid phosphatase 5b (TRACP‐5b). The strengths of associations among biomarkers (ie, pentosidine and bone turnover markers) were then compared. In this study, the data of the Japanese Osteoporosis Intervention Trial‐05 (JOINT‐05) were used. JOINT‐05 was a head‐to‐head, randomized, controlled trial that compared the anti‐fracture efficacy of sequential therapy with teriparatide followed by alendronate and monotherapy with alendronate.^(^ ^21^ ^)^

## 2 Methods

### 2.1 Study design and ethical considerations

In this study, a post hoc analysis was conducted of anonymized data obtained from JOINT‐05, which enrolled 1011 patients from 113 institutes nationwide in Japan between October 2014 and December 2017.^(^ ^21^ ^)^ The study followed the Japanese regional regulations (Clinical Trials Act), which do not require prior review of protocols by the ethics committee and acquisition of informed consent from subjects for observational studies using anonymized data.

### 2.2 Participants

The design and primary results of JOINT‐05 have been reported elsewhere.^(^ ^21^, ^22^ ^)^ In brief, Japanese women aged at least 75 years were eligible for the trial if they had primary osteoporosis and if they were at high risk of fracture. Primary osteoporosis was diagnosed according to the revised 2012 Diagnostic Criteria for Primary Osteoporosis of the Japanese Society for Bone and Mineral Research.^(^ ^23^ ^)^ Patients at high risk of fracture were defined as those who had one of the following: (i) BMD <60% of young adult mean or less than −3.3 standard deviations (SDs); (ii) at least two vertebral fractures in the area from the fourth thoracic vertebra (Th_4_) to the fourth lumbar vertebra (L_4_); (iii) a grade 3 prevalent fracture; or (iv) a past hip fracture.

Patients were excluded from the study if they had secondary osteoporosis due to prespecified conditions; diagnosis of a disease other than osteoporosis that causes bone loss; diagnosis of a disease that affects the strength of the vertebral bodies; history of hypersensitivity; contraindication to any of the study drugs used; serious renal disease, hepatic disease, or cardiac disease; been hospitalized; or history of treatment with teriparatide.

### 2.3 Treatments

Patients were randomly assigned in a 1:1 ratio to receive sequential therapy (once‐weekly subcutaneous injection of teriparatide 56.5 μg for 72 weeks followed by alendronate for 48 weeks) or monotherapy with alendronate for 120 weeks. Alendronate was administered as the following formulations: 5 mg tablet (orally administered once daily), 35 mg tablet or jelly (orally administered once weekly), or 900 μg infusion bag (administered intravenously once every 4 weeks). Nature Made Vitamin D400 supplements were also provided in both arms throughout the entire treatment period.

### 2.4 Baseline measurements

Physical function was evaluated with the timed‐up‐and‐go test (TUG) and the one‐leg standing test with eyes open (OLST). For the TUG, the total time to stand up from a standard chair, walk a distance of 3 m, turn, walk back to the chair, and sit down again was measured. For the OLST, how long patients could stand until their body swayed so much that they felt like they were going to fall, or until their raised leg touched the floor, or the standing leg shifted, whichever occurred first, was measured. The degree of back pain was assessed using a visual analog scale (VAS), ranging from 0 to 100 points, at rest and in motion.

### 2.5 Outcome measures

The thoracic and lumbar vertebrae were imaged in two directions at 0 (baseline), 24, 48, 72, and 120 weeks. For the assessment of prevalent vertebral fractures, anteroposterior and lateral radiographs of the thoracic and lumbar spine were examined by the investigators. They assessed the grade of vertebral fractures from Th_4_ to L_4_ according to a semiquantitative (SQ) technique.^(^ ^24^ ^)^ These assessments were reviewed centrally by one evaluator of the fracture assessment committee blinded to the assigned treatment.

The committee also adjudicated the presence or absence of a new vertebral fracture by comparing radiographs of Th_4_ to L_4_ between baseline and post‐treatment. After the X‐ray films were collected, two evaluators blinded to the assigned treatment reviewed the films independently according to the SQ technique mentioned above. If inconsistencies arose between the evaluators, three evaluators reviewed the films simultaneously. The presence or absence of other fractures, such as nonvertebral fractures and clinical fractures, was assessed by the investigators. Thereafter, three evaluators of the fracture assessment committee reviewed the assessment made by the investigators using the collected X‐ray films.

### 2.6 Measurements of biomarker levels

BMD at the lumbar spine, proximal femur, radius, and second metacarpal bone was measured at 0, 24, 48, 72, and 120 weeks in each institution by dual‐energy X‐ray absorptiometry. Urinary pentosidine levels were measured at 0, 24, 72, and 120 weeks by the ELISA method.^(^ ^20^ ^)^ The correlation coefficient between values obtained from the HPLC method and the ELISA method in the same urine samples was 0.815. Blood samples were obtained at 0, 12, 24, 48, 72, and 120 weeks to measure the serum levels of osteocalcin, P1NP, and TRACP‐5b. LSI Medience Corporation (Tokyo, Japan) analyzed the levels of osteocalcin and P1NP using a fluorometric enzyme immunoassay and an electrochemiluminescence immunoassay, respectively. SB Bioscience Co., Ltd. (Tokyo, Japan) analyzed TRACP‐5b levels using an enzyme immunoassay.

### 2.7 Statistical analysis

Baseline characteristics and laboratory measurements are reported as means with SD or as percentages, and they were compared across quartiles of pentosidine levels by trend tests using generalized linear models.

To investigate the associations between the levels of biomarkers (pentosidine, osteocalcin, P1NP, and TRACP‐5b) and the incidence of fracture, multivariate‐adjusted Poisson regression models were fitted. The following covariates were determined on the basis of the literature^(^ ^9^, ^13^ ^)^ and forced into the models: allocated treatment, age, weight, presence or absence of diabetes mellitus, *T*‐score of BMD, presence or absence of prevalent vertebral fractures, and back pain assessed by the VAS at rest. Baseline levels of biomarkers, as well as their changes from baseline to 24 weeks, were used as risk factors in the Poisson regression analysis. Because the estimated relationships between the incidence of fracture and the levels of biomarkers are potentially nonlinear, and their units of measurement differ, quartile‐specific rate ratios with 95% confidence intervals (CIs) and *p* values were calculated for each biomarker. To explore potential nonlinear relationships further, the spline function and 95% CI of the associations between the urinary pentosidine level and the incidence of vertebral or nonvertebral fracture were also estimated using the generalized additive model. In this analysis, the degrees of freedom were determined by the generalized cross‐validation method.

All reported *p* values are two‐tailed, and *p* < 0.05 was considered to indicate significance. Missing data were handled by complete case analysis. All statistical analyses were performed by academic statisticians using SAS Version 9.4 (SAS Institute, Cary, NC, USA).

## 3 Results

### 3.1 Distribution of urinary pentosidine levels and baseline characteristics

A total of 985 patients were included in the main analysis of JOINT‐05. Of these, 967 patients who had baseline urinary pentosidine measurements were included in this study (Supplemental Fig. S1). Supplemental Fig. S2 shows the histogram of baseline levels of pentosidine. The mean pentosidine level was 44.9 pmol/mL Cr, and the 5th and 95th percentile values were 23.8 and 87.3 pmol/mL Cr, respectively. Table 1 shows the baseline characteristics of the patients divided into four groups according to quartiles of pentosidine levels. The cut‐off values of the pentosidine quartiles were 30, 40, and 50 pmol/mL Cr. The mean ± SD pentosidine level for the first, second, third, and fourth quartile groups was 25.5 ± 4.0 (*n* = 193), 34.9 ± 2.8 (*n* = 301), 44.4 ± 3.0 (*n* = 202), and 70.3 ± 27.9 (*n* = 271) pmol/mL Cr, respectively.

**Table 1:** Baseline Characteristics of Postmenopausal Women With Severe Osteoporosis by Quartile of Penstosidine

	1st quartile (*n* = 193)			2nd quartile (*n* = 301)			3rd quartile (*n* = 202)			4th quartile (*n* = 271)			
	Mean	SD	Missing	Mean	SD	Missing	Mean	SD	Missing	Mean	SD	Missing	Trend *p*
Pentosidine (pmol/mL of Cr)	25.5	4.0	0	34.9	2.8	0	44.4	3.0	0	70.3	27.9	0	—
Age (years)	79.8	3.9	0	81.2	4.5	0	81.8	4.8	0	82.6	4.7	0	<0.01
Age at menopause (years)	49.4	3.9	0	50.0	3.6	0	49.4	4.1	0	48.7	5.5	0	0.01
No. of prevalent vertebral fractures	1.4	1.8	0	1.6	1.9	0	1.8	2.1	0	1.8	1.9	0	0.02
0	40.9%		0	32.2%		0	32.7%		0	26.6%		0	
1	24.4%		0	28.6%		0	24.3%		0	29.5%		0	
2	13.5%		0	15.3%		0	17.3%		0	17.3%		0	
3	9.3%		0	10.0%		0	8.9%		0	11.4%		0	
4	5.2%		0	5.0%		0	6.4%		0	5.5%		0	
5 or more	6.7%		0	9.0%		0	10.4%		0	9.6%		0	
Maximum grade of prevalent vertebral fractures													
Grade 1	10.4%		0	9.3%		0	6.9%		0	10.0%		0	0.97
Grade 2	14.5%		0	18.9%		0	16.3%		0	16.6%		0	0.70
Grade 3	34.2%		0	39.5%		0	44.1%		0	46.9%		0	0.04
History of hip fracture	7.3%		0	10.0%		0	15.3%		0	21.8%		0	<0.01
Prior treatment	55.4%		0	52.5%		0	56.9%		0	53.5%		0	0.82
Prior bisphosphonates	35.2%		1	30.2%		0	27.7%		1	27.3%		1	0.10
Allocated treatment													
Experimental (teriparatide to alendronate)	50.3%			49.2%			46.5%			50.2%			
Control (alendronate to alendronate)	49.7%			50.8%			53.5%			49.8%			
BMD (*T*‐score)	−3.1	1.7	17	−3.1	1.7	22	−3.2	2.0	25	−3.3	1.8	34	0.17
BMD at L_2_ to L_4_ (*T*‐score)	−2.4	1.2	70	−2.3	1.4	126	−2.2	1.6	104	−2.4	1.5	142	0.61
BMD at femoral neck (*T*‐score)	−3.2	0.9	92	−3.0	1.1	147	−3.2	1.0	106	−3.6	1.0	146	<0.01
Osteocalcin (ng/mL)	16.2	7.3	0	17.4	9.6	2	19.0	11.7	0	20.5	13.8	1	<0.01
P1NP (μg/L)	45.2	25.0	0	48.9	30.9	2	56.6	40.9	0	73.4	59.9	0	<0.01
TRACP‐5b (mU/dL)	407.1	166.4	0	456.9	194.8	2	475.1	224.2	0	544.7	254.4	1	<0.01
Comorbidities													
Hypertension	31.1%		0	35.5%		0	38.6%		0	43.9%		0	0.02
Diabetes mellitus	3.6%		0	6.0%		0	10.4%		0	14.0%		0	<0.01
Dyslipidemia	15.0%		0	15.9%		0	12.9%		0	17.7%		0	0.91
Rheumatoid arthritis	0.0%		0	0.7%		0	1.5%		0	1.1%		0	0.17
Osteoarthritis	0.0%		0	0.0%		0	0.5%		0	0.0%		0	0.98
Others	20.2%		0	22.3%		0	27.7%		0	37.6%		0	<0.01
BMI (kg/m^2^)	22.3	3.3	0	22.5	3.5	2	22.3	3.7	1	21.5	4.0	0	0.01
Systolic blood pressure (mmHg)	136.9	21.0	20	137.5	19.4	59	133.3	19.2	27	134.7	18.7	59	0.13
HbA1C (%)	5.8	0.5	0	5.9	0.5	0	6.0	0.8	0	5.9	0.6	0	0.15
LDL cholesterol (mg/dL)	119.0	32.3	0	117.6	33.0	0	114.0	27.4	0	107.5	28.7	0	<0.01
Triglyceride (mg/dL)	125.4	72.1	0	122.6	67.1	0	115.9	55.0	0	110.1	54.4	0	<0.01
eGFR (mL/min/1.73 m^2^)	65.6	14.5	0	64.0	15.7	0	61.9	17.2	0	61.6	19.6	0	<0.01
25OHVD (ng/mL)	18.1	5.6	0	18.2	5.6	2	17.0	5.7	0	16.8	5.9	0	<0.01
MMSE	27.5	2.7	0	27.1	3.3	8	26.0	3.9	3	25.4	5.4	2	<0.01
≥28 or more	56.0%		0	53.2%		8	39.6%		3	38.0%		2	
24–27	38.9%		0	33.9%		8	38.6%		3	42.8%		2	
≤23	5.2%		0	10.3%		8	20.3%		3	18.5%		2	
Timed‐up‐and‐go test (seconds)	11.2	6.1	1	12.9	10.3	7	13.7	8.6	5	15.2	11.3	9	<0.01
One‐leg standing (seconds)	19.0	26.0	1	16.3	23.3	8	15.0	24.9	6	10.4	19.1	9	<0.01

BMD = bone mineral density; BMI = body mass index; eGFR = estimated glomerular filtration rate; HbA1C = glycated hemoglobin; LDL = low‐density lipoproteins; MMSE = mini‐mental state examination; P1NP = procollagen type I amino‐terminal propeptide; TRACP‐5b = tartrate‐resistant acid phosphatase 5b.

The mean age ranged from 79.8 years for the first quartile group to 82.6 years for the fourth quartile group, showing a tendency toward older age in groups with higher pentosidine levels. Hypertension, diabetes mellitus, and other comorbidities were more prevalent in groups with higher pentosidine levels. No significant association was observed between pentosidine quartile level and systolic blood pressure or hemoglobin A1c. The body mass index, low‐density lipoprotein cholesterol level, triglyceride level, estimated glomerular filtration rate (eGFR), and 25‐hydroxy vitamin D level tended to be lower in groups with higher pentosidine levels. Of these baseline values, the correlation between the pentosidine level and eGFR was not significant with the analysis adjusted for age (data not shown).

As shown in Table 1, BMD at the femoral neck was significantly lower in groups with higher pentosidine levels. Serum levels of bone formation markers (P1NP and osteocalcin) and the bone resorption marker (TRACP‐5b) increased in groups with higher pentosidine levels. Cognitive function measured with the mini‐mental state examination and musculoskeletal ambulation disability assessed with the TUG and OLST tended to be worse in the second, third, and fourth quartile groups compared with those in the first quartile group.

Supplemental Table S1 shows the results of logistic regression analyses performed to examine the relationships between pentosidine level and fracture status (presence or absence of prevalent vertebral fracture, grade 3 vertebral fracture, and a history of hip fracture). The pentosidine level was associated with the presence or absence of a history of hip fracture: the adjusted odds ratios for the second, third, and fourth quartile groups to the first quartile group were 1.16 (95% CI 0.58–2.29, *p* = 0.68), 1.79 (95% CI 0.89–3.59, *p* = 0.10), and 2.54 (95% CI 1.32–4.87, *p* = 0.01), respectively.

### 3.2 Effects of osteoporosis treatment on urinary pentosidine level

Pentosidine measurements during the treatment period were obtained from 741 patients at week 24, 616 at week 72, and 521 at week 120. Supplemental Table S2 shows the least square means of pentosidine levels in the experimental (sequential therapy) and control (monotherapy) groups. At week 120, there was no difference in pentosidine levels between the treatment groups (40.20 versus 40.23 pmol/mL Cr, *p* = 0.99).

### 3.3 Associations between baseline levels of biomarkers and the incidence of fracture

Analysis populations for morphometric and vertebral and nonvertebral fracture were described in Supplemental Fig. S1. At week 120, 530 patients were followed up for fracture status (of these, 23 died). The remaining 437 patients were not followed up. The main reasons for losses to follow‐up were related to the patients' personal requirements or safety. Vertebral fracture occurred in 137 patients and nonvertebral fracture in 42. The number of patients who had vertebral fractures (annual incidence) by pentosidine quartile was 20 (0.914 per person‐year), 43 (0.131 per person‐year), 28 (0.127 per person‐year), and 46 (0.160 per person‐year) for the first, second, third, and fourth quartile groups, respectively. The number of patients who suffered nonvertebral fracture (annual incidence) was 4 (0.012 per person‐year), 13 (0.033 per person‐year), 9 (0.031 per person‐year), and 16 (0.042 per person‐year), respectively.

Tables 2 and 3 show the results of the Poisson regression analyses performed to examine the associations between the baseline level of each biomarker (pentosidine, osteocalcin, P1NP, or TRACP‐5b) and the incidences of vertebral and nonvertebral fractures, respectively. In terms of the incidence of vertebral fracture, the rate ratios for the second, third, and fourth quartile groups of pentosidine levels compared with the first quartile group were 1.65 (95% CI 0.99–2.75, *p* = 0.06), 1.51 (95% CI 0.87–2.61, *p* = 0.14), and 1.69 (95% CI 1.01–2.83, *p* = 0.05), respectively. The corresponding rate ratios in terms of nonvertebral fracture were 3.07 (95% CI 0.88–10.70, *p* = 0.08), 2.34 (95% CI 0.61–8.95, *p* = 0.22), and 3.95 (95% CI 1.14–13.67, *p* = 0.03), respectively. Similar trends were observed when patients were analyzed separately by allocated treatment (Tables S3 and S4).

**Table 2:** Associations Between Urinary Pentosidine and Bone Turnover Markers and the Incidence of Morphometric Vertebral Fracture^a^

	Pentosidine				Osteocalcin				P1NP				TRACP‐5b			
	Rate ratio	95% CI		*p*	Rate ratio	95% CI		*p*	Rate ratio	95% CI		*p*	Rate ratio	95% CI		*p*
Category of each marker																
1st quartile	Ref				Ref				Ref				Ref			
2nd quartile	1.65	0.99	2.75	0.06	0.79	0.49	1.25	0.31	1.68	1.05	2.68	0.03	1.94	1.16	3.25	0.01
3rd quartile	1.51	0.87	2.61	0.14	1.12	0.73	1.72	0.60	1.60	0.98	2.62	0.06	1.80	1.05	3.07	0.03
4th quartile	1.69	1.01	2.83	0.05	1.20	0.78	1.85	0.42	2.05	1.27	3.29	<0.01	2.38	1.43	3.97	<0.01
Allocated to experimental group	0.84	0.61	1.15	0.27	0.84	0.61	1.16	0.30	0.80	0.58	1.11	0.18	0.80	0.58	1.11	0.18
Age (+10 years)	1.11	0.80	1.56	0.53	1.13	0.81	1.58	0.46	1.11	0.80	1.55	0.52	1.12	0.80	1.55	0.51
Weight (+10 kg)	0.81	0.66	1.00	0.05	0.80	0.65	0.98	0.03	0.78	0.63	0.96	0.02	0.84	0.68	1.03	0.09
Diabetes mellitus	0.70	0.36	1.39	0.31	0.73	0.37	1.43	0.36	0.70	0.35	1.38	0.31	0.69	0.35	1.37	0.29
BMD (+1 *T*‐score)	1.03	0.94	1.12	0.57	1.03	0.94	1.13	0.53	1.04	0.95	1.13	0.44	1.03	0.94	1.12	0.56
Prevalent vertebral fracture	1.18	1.12	1.26	<0.01	1.18	1.12	1.26	<0.01	1.18	1.12	1.26	<0.01	1.19	1.12	1.26	<0.01
Back pain (+10% of maximum)	1.09	1.03	1.15	<0.01	1.09	1.03	1.15	<0.01	1.08	1.02	1.14	0.01	1.08	1.02	1.14	0.01

BMD = bone mineral density; CI = confidence interval; P1NP = procollagen type I amino‐terminal propeptide; TRACP‐5b = tartrate‐resistant acid phosphatase 5b.

^a^ Multivariate Poisson regression.

**Table 3:** Associations Between Quartiles of Urinary Pentosidine and Bone Turnover Markers and Incidence of Nonvertebral Fracture^a^

	Pentosidine				Osteocalcin				P1NP				TRACP‐5b			
	Rate ratio	95% CI		*p*	Rate ratio	95% CI		*p*	Rate ratio	95% CI		*p*	Rate ratio	95% CI		*p*
Category of each marker																
1st quartile	Ref				Ref				Ref				Ref			
2nd quartile	3.07	0.88	10.70	0.08	1.16	0.46	2.95	0.76	2.30	0.96	5.54	0.06	1.00	0.39	2.53	0.99
3rd quartile	2.34	0.61	8.95	0.22	1.23	0.50	3.03	0.65	1.39	0.48	4.01	0.54	1.12	0.45	2.78	0.80
4th quartile	3.95	1.14	13.67	0.03	1.99	0.86	4.63	0.11	1.96	0.76	5.08	0.17	1.82	0.78	4.26	0.17
Allocated to experimental group	1.55	0.85	2.84	0.15	1.56	0.85	2.85	0.15	1.50	0.82	2.75	0.19	1.52	0.83	2.79	0.17
Age (+10 years)	1.56	0.80	3.07	0.20	1.66	0.86	3.21	0.13	1.63	0.85	3.13	0.14	1.63	0.85	3.15	0.14
Weight (+10 kg)	1.13	0.78	1.63	0.53	1.10	0.75	1.62	0.61	1.07	0.73	1.58	0.74	1.16	0.79	1.70	0.45
Diabetes mellitus	0.91	0.32	2.59	0.86	1.04	0.37	2.93	0.94	1.04	0.37	2.94	0.94	0.97	0.34	2.77	0.96
BMD (+1 *T*‐score)	0.95	0.79	1.14	0.58	0.97	0.81	1.16	0.73	0.96	0.80	1.15	0.62	0.95	0.79	1.14	0.58
Prevalent vertebral fracture	1.29	1.16	1.44	<0.01	1.31	1.17	1.46	<0.01	1.28	1.15	1.43	<0.01	1.30	1.16	1.45	<0.01
Back pain (+10% of maximum)	0.90	0.79	1.02	0.11	0.90	0.79	1.02	0.10	0.90	0.79	1.03	0.12	0.90	0.79	1.02	0.10

BMD = bone mineral density; CI = confidence interval; P1NP = procollagen type I amino‐terminal propeptide; TRACP‐5b = tartrate‐resistant acid phosphatase 5b.

^a^ Multivariate Poisson regression.

Of the bone turnover markers, the serum TRACP‐5b level was highly associated with the incidence of fracture (Tables 2 and 3). When pentosidine and TRACP‐5b levels were included simultaneously in the Poisson regression model, the TRACP‐5b level remained a significant risk factor for vertebral fracture (Supplemental Table S5), whereas the pentosidine level remained a significant factor for nonvertebral fracture (Table S6).

**Fig. 1:**
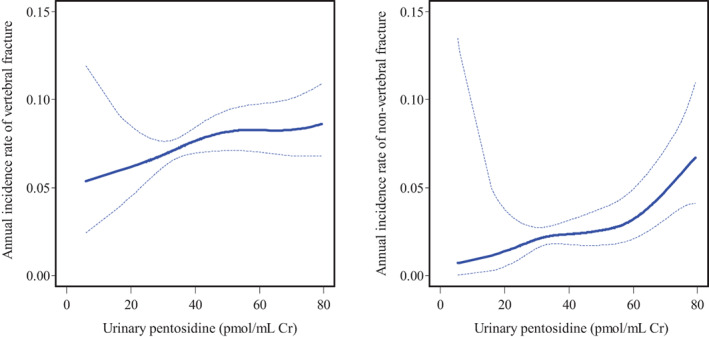
Spline curves of the urinary pentosidine level for annual incidence rates of morphometric vertebral fracture and nonvertebral fracture.

The relationships between urinary pentosidine levels and the incidences of vertebral and nonvertebral fractures were further examined by the generalized additive model (Fig. 1). As shown graphically, the relationships were approximately linear for both fractures without any indication of the presence of a threshold.

### 3.4 Associations between changes in biomarker levels at 24 weeks and the incidence of fracture

Tables 4 and 5 show the results of the Poisson regression analyses performed to examine the associations between the changes in biomarker levels and the incidences of vertebral and nonvertebral fractures, respectively. Change in pentosidine levels was not associated with the incidence of vertebral or nonvertebral fracture, whereas reductions in osteocalcin, P1NP, and TRACP‐5b levels were associated with higher incidences of vertebral and nonvertebral fractures. The rate ratios per 1 SD ranged from 1.16 to 2.21.

**Table 4:** Associations Between Changes in Urinary Pentosidine and Bone Turnover Markers and the Incidence of Morphometric Vertebral Fracture^a^

	Pentosidine				Osteocalcin				P1NP				TRACP‐5b			
	Rate ratio	95% CI		*p*	Rate ratio	95% CI		*p*	Rate ratio	95% CI		*p*	Rate ratio	95% CI		*p*
Changes at 24 weeks (+1 SD)	1.07	0.92	1.24	0.40	1.24	1.02	1.51	0.03	1.19	0.98	1.45	0.08	1.16	0.99	1.36	0.07
Allocated to experimental group	1.15	0.83	1.60	0.40	1.50	1.01	2.22	0.04	1.35	0.94	1.93	0.10	1.26	0.91	1.75	0.17
Age (+10 years)	1.24	0.88	1.75	0.22	1.14	0.81	1.60	0.46	1.14	0.81	1.60	0.44	1.14	0.81	1.60	0.46
Weight (+10 kg)	0.80	0.65	0.99	0.04	0.80	0.64	0.99	0.04	0.79	0.64	0.98	0.03	0.79	0.64	0.97	0.03
Diabetes mellitus	0.81	0.41	1.60	0.55	0.78	0.39	1.53	0.46	0.84	0.42	1.64	0.60	0.82	0.41	1.61	0.56
BMD (+1 *T*‐score)	1.05	0.95	1.15	0.34	1.04	0.95	1.13	0.43	1.03	0.94	1.13	0.47	1.02	0.94	1.12	0.60
Prevalent vertebral fracture	1.19	1.12	1.27	<0.01	1.18	1.11	1.25	<0.01	1.18	1.11	1.25	<0.01	1.17	1.10	1.24	<0.01
Back pain (+10% of maximum)	1.08	1.02	1.14	0.01	1.08	1.02	1.14	0.01	1.08	1.03	1.14	<0.01	1.08	1.03	1.14	<0.01

BMD = bone mineral density; CI = confidence interval; P1NP = procollagen type I amino‐terminal propeptide; TRACP‐5b = tartrate‐resistant acid phosphatase 5b.

^a^ Multivarieate Poisson regression.

**Table 5:** Associations Between Changes in Urinary Pentosidine and Bone Turnover Markers and the Incidence of Nonvertebral Fracture^a^

	Pentosidine				Osteocalcin				P1NP				TRACP‐5b			
	Rate ratio	95% CI		*p*	Rate ratio	95% CI		*p*	Rate ratio	95% CI		*p*	Rate ratio	95% CI		*p*
Change at 24 weeks (+1 SD)	1.38	0.91	2.09	0.13	1.57	1.11	2.23	0.01	2.21	1.61	3.04	<0.01	1.44	1.04	1.99	0.03
Allocated to experimental group	0.77	0.41	1.44	0.41	1.22	0.57	2.63	0.61	1.53	0.74	3.19	0.25	0.83	0.44	1.58	0.58
Age (+10 years)	1.31	0.65	2.64	0.46	1.24	0.61	2.53	0.54	1.39	0.67	2.87	0.38	1.24	0.61	2.52	0.56
Weight (+10 kg)	1.05	0.70	1.56	0.82	1.07	0.71	1.59	0.76	1.13	0.74	1.72	0.58	1.04	0.70	1.57	0.83
Diabetes mellitus	1.28	0.45	3.63	0.64	1.24	0.44	3.50	0.69	1.45	0.51	4.10	0.49	1.37	0.48	3.89	0.56
BMD (+1 *T*‐score)	0.93	0.78	1.11	0.44	0.93	0.78	1.12	0.44	0.91	0.75	1.10	0.32	0.91	0.75	1.10	0.32
Prevalent vertebral fracture	1.24	1.10	1.39	<0.01	1.24	1.11	1.40	<0.01	1.21	1.07	1.36	<0.01	1.22	1.08	1.37	<0.01
Back pain (+10% of maximum)	0.90	0.79	1.04	0.15	0.91	0.79	1.04	0.17	0.94	0.82	1.08	0.40	0.92	0.80	1.06	0.24

BMD = bone mineral density; CI = confidence interval; P1NP = procollagen type I amino‐terminal propeptide; TRACP‐5b = tartrate‐resistant acid phosphatase 5b.

^a^ Multivariate Poisson regression.

## 4 Discussion

In 967 postmenopausal women with severe osteoporosis treated with teriparatide or alendronate, the risks of vertebral and nonvertebral fractures increased depending on the baseline urinary pentosidine level. The association between the pentosidine level and vertebral fracture was similar to or somewhat weaker than that between serum levels of bone turnover markers (TRACP‐5b or P1NP) and vertebral fracture. For nonvertebral fracture, the pentosidine level was the strongest risk factor. Analyses using the Poisson regression models and generalized additive models showed an approximately linear relationship between the pentosidine level and the incidence of fracture, without any notable threshold.

Between the treatment groups, there were no differences in the association of the baseline pentosidine level with fracture incidence. Furthermore, no association was observed between the changes in pentosidine levels at week 24 and fracture incidence in either treatment group. In the previous animal experiments, a decrease in the pentosidine level was noted after the administration of teriparatide to monkeys, which was thought to reflect the osteogenic effect of teriparatide.^(^ ^25^ ^)^ This interpretation is inconsistent with the present result showing no difference between the treatment groups. Although there are few reports showing the effect of drug treatment on the urinary pentosidine level, the present study suggests that pentosidine may be a biomarker not specific to any therapeutic agent for osteoporosis.

Because this was a post hoc analysis of a clinical trial, unlike the preceding Health ABC Study,^(^ ^15^ ^)^ OFFLEY Study,^(^ ^16^ ^)^ Nagano Cohort Study,^(^ ^13^, ^17^, ^18^ ^)^ and JOINT‐04,^(^ ^14^ ^)^ the analyzed population consisted of patients aged as old as 80 years on average, being treated for osteoporosis, and at an increased risk of fracture, with an actual fracture incidence within 1 year of at least 10%. The pathology of osteoporosis differs by age. Previous epidemiological studies identified the pentosidine level as a risk factor for fracture in postmenopausal women or patients with type 2 diabetes mellitus.^(^ ^13^, ^15^, ^16^, ^17^, ^18^ ^)^ In contrast, the present study identified the risk factors for fracture in patients with osteoporosis, especially those considered by clinicians to be at a high risk of fracture. The significant risk factors for vertebral fracture were the pentosidine level, weight, prevalent vertebral fracture, and back pain. For nonvertebral fracture, the significant risk factors were the pentosidine level, weight, and prevalent vertebral fracture. Of the bone turnover markers, baseline serum levels of P1NP and TRACP‐5b, as well as changes in serum levels of osteocalcin, P1NP, and TRACP‐5b, were shown to be associated with the incidences of fractures. Age and BMD, which are established risk factors for fracture, were not significant in the present study. This is probably because the JOINT‐05 restricted its population to elderly patients with low BMD. Many patients being treated for osteoporosis are elderly and have low BMD. If the risk factors for fracture in this population are different from those in populations commonly examined in the epidemiological studies, such differences may suggest that the existing tools including FRAX^(^ ^3^ ^)^ and QFracture^(^ ^4^ ^)^ are suboptimal for risk assessment of patients on treatment.

The present study suggests that bone quality and bone metabolism markers are useful as biomarkers for identifying high‐risk populations for fracture in patients on treatment. In the assessment of fracture risk during treatment, information regarding prevalent vertebral fracture and fracture history would be the most important. Levels of pentosidine or bone metabolism markers will then be measured according to the specific objective. The pentosidine level was shown to be strongly associated with nonvertebral fracture, in particular, which would be attributed to the involvement of pentosidine in the decline of motor function (eg, TUG and OLST) and reflects the increased risk of nonvertebral fractures owing to falls and trauma. A previous study suggested a two‐step algorithm to identify high‐risk patients using pentosidine.^(^ ^14^ ^)^ In the two‐step algorithm, (i) 10‐year fracture risk of a woman is evaluated using one of the conventional risk assessment tools^(^ ^3^, ^9^ ^)^ and the woman is classified into the low‐ or high‐risk group based on the 10‐year risk and a cut‐off value of 15%, and (ii) the low‐risk group is further subcategorized according to the pentosidine level with a cut‐off value of 50 pmol/mg of Cr. Our study supported that this algorithm works well even for risk assessment of patients receiving medications for osteoporosis, although further research to validate fracture risk assessment tools in the setting of treated patients is needed.^(^ ^2^ ^)^

Several limitations warrant mention. First, the follow‐up period of JOINT‐05 was limited to 120 weeks, and the present data included only 137 and 42 cases of vertebral and nonvertebral fractures, respectively. Thus, it is inherently impossible to estimate 10‐year fracture probability on the basis of the urinary pentosidine level or to develop complicated regression models with high‐dimensional covariates. However, the statistical power of the Poisson regression analysis was sufficient even for nonvertebral fracture, since the observed association between the urinary pentosidine level and nonvertebral fracture was strong. Second, the conclusion was mainly based on the whole data set, in which the data obtained from two treatment groups were combined, although pharmacological treatments affect BMD and levels of bone turnover markers during follow‐up. The sample size of each treatment group was not large in JOINT‐05, and, therefore, the results with separate data sets by allocated treatment are presented only as Online Resources (Tables S1–S3). Finally, BMD was measured at the lumbar spine, proximal femur, radius, or second metacarpal bone in each institution. The analytical methods for BMD were not standardized across the institutions, and this is a major limitation of this study.

In conclusion, the urinary pentosidine level was associated with fracture risk in patients with severe osteoporosis receiving teriparatide or alendronate.

## Disclosures

ST has received lecture fees from Bayer Yakuhin, Amgen Astellas BioPharma KK, and the Research Institute of Healthcare Data Science. He has received consultation fees and outsourcing fees from Daiichi Sankyo Company, Limited, Boehringer Ingelheim, Satt Co., Ltd., and the Public Health Research Foundation. He has received research grants from the Japan Agency for Medical Research and Development, the Japanese Ministry of Health Labour and Welfare, the Japanese Ministry of Education, Science, and Technology, and Novo Nordisk. He is currently engaged in a research project for the Japan Agency for Medical Research and Development. MS has received lecture fees or grants outside the submitted work from Amgen Inc., Asahi Kasei Pharma Corp., Astellas Pharma Inc., Chugai Pharmaceutical Co., Ltd., Daiichi Sankyo Co., Ltd., Eisai Co., Ltd., Eli Lilly Japan Co., Ltd., Mochida Pharma Co., Ltd., Ono Pharmaceutical Co., Ltd., Taisho Pharmaceutical Co., Ltd., and Teijin Pharma Ltd. HH has received lecture fees or grants outside the submitted work from Amgen Inc., Asahi Kasei Pharma Corp., Astellas Pharma Inc., Chugai Pharmaceutical Co., Ltd., Daiichi Sankyo Co., Ltd., Eisai Co., Ltd., Eli Lilly Japan Co., Ltd., Mitsubishi Tanabe Pharma Corp., Mochida Pharma Co., Ltd., Ono Pharmaceutical Co., Ltd., Pfizer Japan Inc., Taisho Pharmaceutical Co., Ltd., Teijin Pharma Ltd., and UCB Japan Co., Ltd. TN has received personal fees and other fees from Asahi Pharma Corp., Teijin Pharma Ltd., Daiichi‐Sankyo Pharma Co., Ltd., UCB Japan Co., Ltd., Amgen Inc., Astellas Pharma Inc., Chugai Pharma Co., Ltd., and Merck & Co., Inc. HO declares no competing interests. TSo has received research grants from Astellas Pharma Inc., Eisai Co., Ltd., Daiichi‐Sankyo Co., Ltd., Chugai Pharmaceutical Co., Ltd., and Eli Lilly Japan Co., Ltd., as well as consulting and/or lecture fees from Asahi Kasei Pharma Corp., MSD Co., Ltd., and Daiichi‐Sankyo Co., Ltd. TN has received personal fees and other from Asahi Pharma Corp., Teijin Pharma Ltd., Daiichi‐Sankyo Pharma Co., Ltd., UCB Japan Co., Ltd., Amgen Inc., Astellas Pharma Inc., and Chugai Pharma Co., Ltd., as well as personal fees and other from Merck & Co., Inc. KT declares no competing interests. YM declares no competing interests. TSu has received research grants from Asahi Kasei Pharma Corp., Astellas Pharma Inc., Taisho Pharmaceutical Co., Ltd., Takeda Pharmaceutical Co., Ltd., Pfizer Japan Inc., and Teijin Pharma Co., Ltd., as well as consulting fees from Kissei Pharmaceutical Co., Ltd., Shimadzu Corp., and Takeda Pharmaceutical Co., Ltd. SS has received consulting fees, speaking fees, and/or honoraria from Amgen Inc., Asahi Kasei Pharma Corp., Astellas Pharma Inc., Chugai Pharmaceutical Co., Ltd., Daiichi Sankyo Co., Ltd., Eisai Co., Ltd., Eli Lilly Japan Co., Ltd., Mochida Pharma Co., Ltd., Ono Pharmaceutical Co., Ltd., Teijin Pharma Ltd., and UCB Japan Co., Ltd. SM declares no competing interests.

## Author Contributions

**Shiro Tanaka:** Conceptualization; formal analysis; writing – original draft. **Satoshi Soen:** Investigation; project administration; writing – review and editing. **Toshitsugu Sugimoto:** Funding acquisition; investigation; project administration; writing – review and editing. **Yuji Mitomo:** Formal analysis; writing – review and editing. **Kaito Takahashi:** Formal analysis; writing – review and editing. **Satoshi Mori:** Investigation; project administration; writing – review and editing. **Hiroshi Hagino:** Investigation; project administration; writing – review and editing. **Mitsuru Saito:** Conceptualization; writing – original draft. **Teruki Sone:** Investigation; project administration; writing – review and editing. **Hiroaki Ohta:** Investigation; project administration; writing – review and editing. **Toshitaka Nakamura:** Investigation; project administration; writing – review and editing.

### 1 Peer Review

The peer review history for this article is available at https://publons.com/publon/10.1002/jbm4.10673.

## Supporting information


**Supplemental Fig. S1.** Flowchart of the patients included in the analysis.
**Supplemental Fig. S2.** Histogram of baseline urinary pentosidine levels.
**Supplemental Table S1.** Associations Between Quartile of Urinary Pentosidine Levels and Prevalent Fracture at Baseline
**Supplemental Table S2.** Comparisons of Urinary Pentosidine Levels Over 120 Weeks Between the Treatment Groups
**Supplemental Table S3.** Associations Between Quartile of Urinary Pentosidine Levels and the Incidence of Morphometric Vertebral Fracture by Treatment Group
**Supplemental Table S4.** Associations Between Quartile of Urinary Pentosidine Levels and the Incidence of Nonvertebral Fracture by Treatment Group
**Supplemental Table S5.** Associations Between Quartile of Urinary Pentosidine Levels and Serum TRACP‐5b Levels and the Incidence of Vertebral Fracture
**Supplemental Table S6.** Associations Between Quartiles of Urinary Pentosidine Levels and Serum TRACP‐5b Levels and the Incidence of Nonvertebral Fracture

## Data Availability

The data that support the findings of this study are available from the corresponding author upon reasonable request.
