# Supplementary material for: Association of Urinary Pentosidine Levels With the Risk of Fractures in Patients With Severe Osteoporosis: The Japanese Osteoporosis Intervention Trial‐05 (JOINT‐05)
Source: JBMR Plus. 2022 Aug 31;6(10):e10673. doi: 10.1002/jbm4.10673 (PMC9549726; doi:10.1002/jbm4.10673)
Supplement: Supplementary file 1 — Supplemental Fig. S1. Flowchart of the patients included in the analysis. Supplemental Fig. S2. Histogram of baseline urinary pentosidine levels. Supplemental Table S1. Associations Between Quartile of Urinary Pentosidine Levels and Prevalent Fracture at Baseline Supplemental Table S2. Comparisons of Urinary Pentosidine Levels Over 120 Weeks Between the Treatment Groups Supplemental Table S3. Associations Between Quartile of Urinary Pentosidine Levels and the Incidence of Morphometric Vertebral Fracture by Treatment Group Supplemental Table S4. Associations Between Quartile of Urinary Pentosidine Levels and the Incidence of Nonvertebral Fracture by Treatment Group Supplemental Table S5. Associations Between Quartile of Urinary Pentosidine Levels and Serum TRACP‐5b Levels and the Incidence of Vertebral Fracture Supplemental Table S6. Associations Between Quartiles of Urinary Pentosidine Levels and Serum TRACP‐5b Levels and the Incidence of Nonvertebral Fracture [file JBM4-6-e10673-s001.pdf]

Supplementary Figure 1. Flowchart of the patients included in the analysis

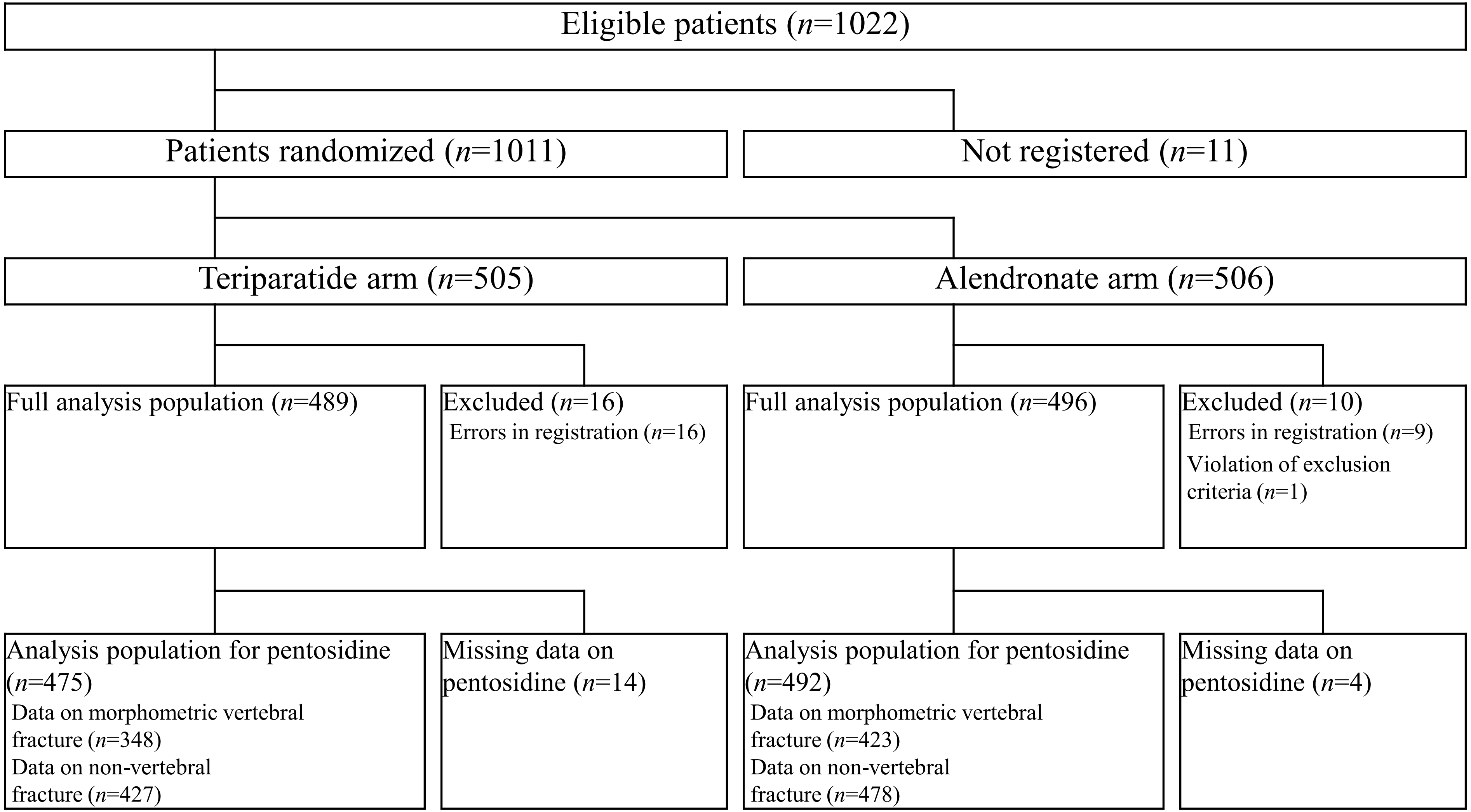

Supplementary Figure 2. Histogram of baseline urinary pentosidine level

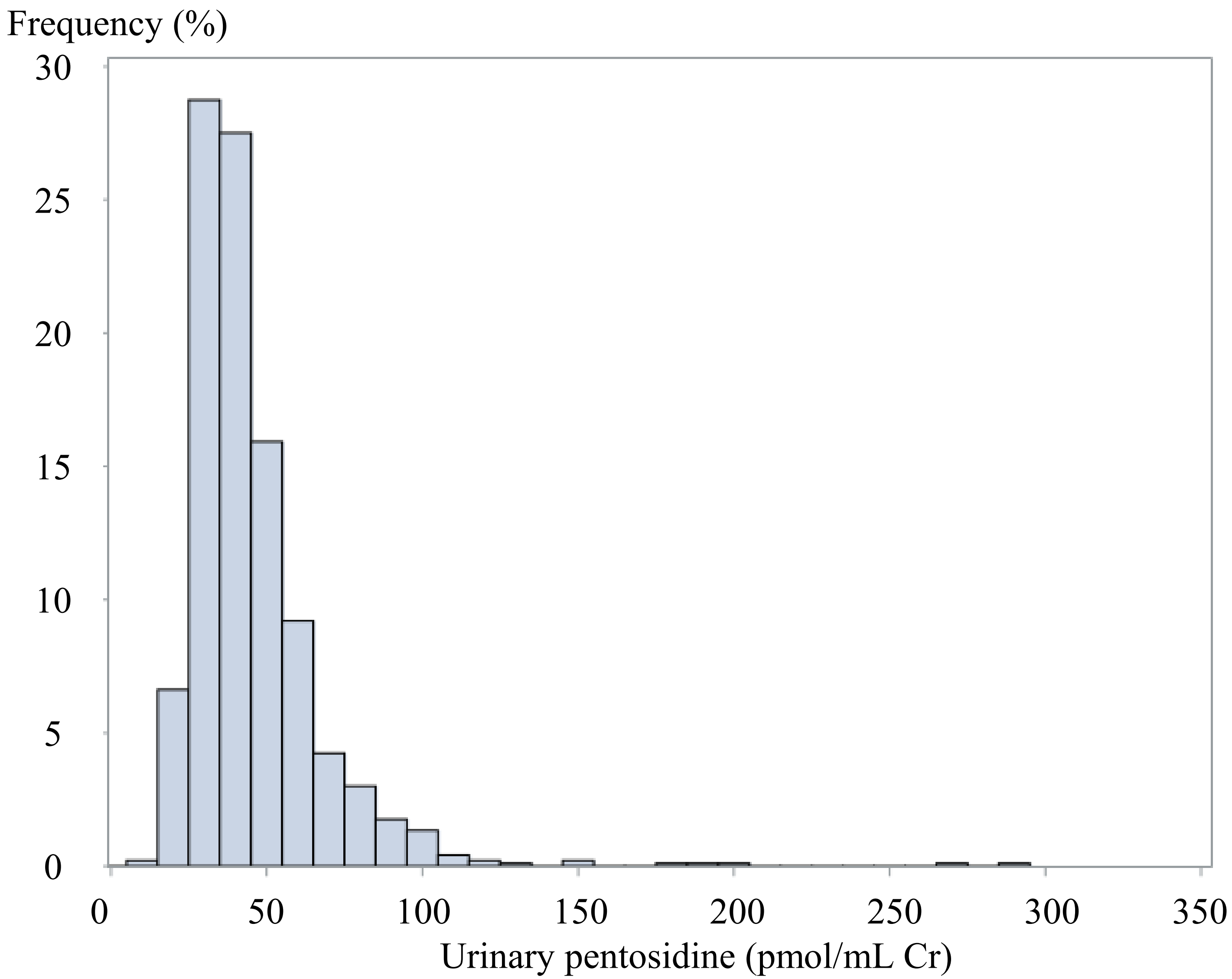

Supplementary Table 1. Associations between quartile of urinary pentosidine levels and prevalent fracture at baseline

	Prevalent morphometric vertebral fracture				Prevalent grade 3 vertebral fracture				History of hip fracture			
	Odds ratio	95% CI		p	Odds ratio	95% CI		p	Odds ratio	95% CI		p
Corrected pentosidine												
1st quartile	Ref				Ref				Ref			
2nd quartile	1.14	0.76	1.70	0.52	1.10	0.74	1.63	0.65	1.16	0.58	2.29	0.68
3rd quartile	1.28	0.82	1.99	0.27	1.33	0.86	2.06	0.20	1.79	0.89	3.59	0.10
4th quartile	1.30	0.85	1.97	0.22	1.34	0.88	2.03	0.17	2.54	1.32	4.87	0.01
Age (+10 y)	1.21	0.89	1.65	0.21	1.55	1.14	2.11	0.01	2.06	1.34	3.18	<0.01
Weight (+10 kg)	0.85	0.72	1.01	0.07	0.98	0.83	1.16	0.84	0.98	0.77	1.24	0.85
Diabetes mellitus	1.01	0.61	1.67	0.98	1.07	0.65	1.77	0.79	2.84	1.62	4.98	<0.01
BMD (+1 T-score)	0.99	0.91	1.07	0.78	0.98	0.91	1.06	0.68	1.03	0.92	1.15	0.62
Back pain (+10% of maximum)	1.04	0.99	1.10	0.14	1.05	1.00	1.11	0.06	0.93	0.86	1.01	0.10

Abbreviations, CI: confidence interval, BMD: bone mineral density

*Multivariate logistic regression using baseline data

Supplementary Table 2. Comparisons of urinary pentosidine levels over 120 weeks between the treatment groups

		Experimental group				Control group							
		LS mean	95% CI		N	LS mean	95% CI		N	Difference	95% CI		p
Corrected pentosidine	0 weeks	45.71	43.80	47.62	475	44.17	42.29	46.04	492				
(pmol/mL)	24 weeks	45.71	43.56	47.86	335	43.48	41.49	45.48	406	-2.23	-5.16	0.70	0.14
	72 weeks	42.88	40.54	45.21	261	41.40	39.32	43.48	355	-1.47	-4.60	1.66	0.36
	120 weeks	40.20	37.73	42.68	219	40.23	38.04	42.42	302	0.02	-3.28	3.33	0.99

Abbreviations, LS means: least square means, CI: confidence interval

*Linear mixed models

Supplementary Table 3. Associations between quartile of urinary pentosidine levels and the incidence of morphometric vertebral fracture by treatment group

	Experimental group (N=348)				Control group (N=423)			
	Rate ratio	95% CI		p	Rate ratio	95% CI		p
Pentosidine								
1st quartile	Ref				Ref			
2nd quartile	2.20	0.97	4.98	0.06	1.34	0.69	2.61	0.39
3rd quartile	2.00	0.84	4.76	0.12	1.40	0.69	2.83	0.35
4th quartile	1.21	0.50	2.89	0.67	2.05	1.08	3.91	0.03
Age (+10 y)	1.10	0.62	1.95	0.74	1.16	0.77	1.75	0.47
Weight (+10 kg)	0.80	0.58	1.11	0.18	0.83	0.63	1.09	0.18
Diabetes mellitus	0.83	0.30	2.32	0.73	0.57	0.23	1.43	0.23
BMD (+1 T-score)	1.10	0.94	1.28	0.22	0.97	0.87	1.08	0.61
Prevalent vertebral fracture	1.22	1.10	1.35	<0.01	1.16	1.08	1.25	<0.01
Back pain (+10% of maximum)	1.09	0.99	1.20	0.08	1.07	1.00	1.15	0.04

Abbreviations, CI: confidence interval, BMD: bone mineral density

*Multivariate Poisson regression

Supplementary Table 4. Associations between quartile of urinary pentosidine levels and the incidence of non-vertebral fracture by treatment group

	Experimental group (N=427)				Control group (N=478)			
	Rate ratio	95% CI		p	Rate ratio	95% CI		p
Pentosidine								
1st quartile	Ref				Ref			
2nd quartile	5.47	0.68	44.13	0.11	1.63	0.32	8.23	0.55
3rd quartile	4.35	0.47	39.91	0.19	1.38	0.24	7.81	0.72
4th quartile	5.68	0.70	46.29	0.10	3.26	0.68	15.59	0.14
Age (+10 y)	1.52	0.55	4.22	0.42	1.94	0.74	5.06	0.18
Weight (+10 kg)	0.84	0.48	1.47	0.54	1.70	0.97	3.00	0.07
Diabetes mellitus	1.47	0.43	5.02	0.54	0.36	0.05	2.81	0.33
BMD (+1 T-score)	0.94	0.71	1.23	0.64	0.95	0.74	1.23	0.70
Prevalent vertebral fracture	1.42	1.20	1.68	<0.01	1.21	1.04	1.41	0.02
Back pain (+10% of maximum)	0.80	0.64	1.00	0.05	0.94	0.80	1.11	0.48

Abbreviations, CI: confidence interval, BMD: bone mineral density

*Multivariate Poisson regression

Supplementary Table 5.Associations between quartile of urinary pentosidine levels and serum TRACP 5b levels and the incidence of vertebral fracture

	Rate ratio	95% CI		p
Pentosidine				
1st quartile	Ref			
2nd quartile	1.56	0.93	2.61	0.09
3rd quartile	1.50	0.86	2.59	0.15
4th quartile	1.54	0.91	2.60	0.11
TRACP 5b				
1st quartile	Ref			
2nd quartile	1.90	1.13	3.19	0.02
3rd quartile	1.74	1.02	2.98	0.04
4th quartile	2.28	1.36	3.83	<0.01
Allocated to experimental group	1.25	0.91	1.72	0.17
Age (+10 y)	1.10	0.78	1.53	0.59
Weight (+10 kg)	0.85	0.69	1.04	0.12
Diabetes mellitus	0.65	0.33	1.29	0.22
BMD (+1 T-score)	1.03	0.95	1.13	0.45
Prevalent vertebral fracture	1.19	1.12	1.26	<0.01
Back pain (+10% of maximum)	1.08	1.02	1.14	0.01

Abbreviations, CI: confidence interval, TRACP 5b: tartrate-resistant acid phosphatase 5b, BMD: bone mineral density

*Multivariate Poisson regression

Supplementary Table 6. Associations between quartiles of urinary pentosidine levels and serum TRACP 5b levels and the incidence of non-verteb

	Rate ratio	95% CI		p
Pentosidine				
1st quartile	Ref			
2nd quartile	2.96	0.85	10.37	0.09
3rd quartile	2.28	0.59	8.78	0.23
4th quartile	3.53	1.00	12.49	0.05
TRACP 5b				
1st quartile	Ref			
2nd quartile	0.96	0.37	2.45	0.93
3rd quartile	1.07	0.42	2.68	0.89
4th quartile	1.49	0.63	3.56	0.37
Allocated to experimental group	0.66	0.36	1.21	0.18
Age (+10 y)	1.52	0.77	2.99	0.23
Weight (+10 kg)	1.16	0.79	1.68	0.45
Diabetes mellitus	0.84	0.29	2.40	0.74
BMD (+1 T-score)	0.96	0.80	1.15	0.65
Prevalent vertebral fracture	1.30	1.16	1.45	<0.01
Back pain (+10% of maximum)	0.90	0.79	1.02	0.11

Abbreviations, CI: confidence interval, TRACP 5b: tartrate-resistant acid phosphatase 5b, BMD: bone mineral density

*Multivariate Poisson regression
